# Age-Specific Outcomes of Bioprosthetic vs. Mechanical Aortic Valve Replacement: Balancing Reoperation Risk with Anticoagulation Burden

**DOI:** 10.3390/jcdd11070227

**Published:** 2024-07-18

**Authors:** Fatimah A. Alhijab, Latifa A. Alfayez, Essam Hassan, Monirah A. Albabtain, Ismail M. Elnaggar, Khaled A. Alotaibi, Adam I. Adam, Claudio Pragliola, Huda H. Ismail, Amr A. Arafat

**Affiliations:** 1 Adult Cardiac Surgery Department, Prince Sultan Cardiac Center, Riyadh 12233, Saudi Arabia; fatimah.alhijab.2013@gmail.com (F.A.A.); emousa.2001@gmail.com (E.H.); ismailelnaggar@kasralainy.edu.eg (I.M.E.); dr.kalotaibi@live.com (K.A.A.); amohamed@pscc.med.sa (A.I.A.); cpraglioloa@pscc.med.sa (C.P.); hismail@pscc.med.sa (H.H.I.); 2 Cardiac Research Department, Prince Sultan Cardiac Center, Riyadh 12233, Saudi Arabia; laalfayez@pscc.med.sa (L.A.A.); muneera_2004@yahoo.com (M.A.A.); 3 Cardiothoracic Surgery Department, Cairo University, Cairo 11562, Egypt; 4 Cardiothoracic Surgery Department, Tanta University, Tanta 31111, Egypt

**Keywords:** aortic valve replacement, bioprosthetic valves, mechanical valves, valve surgery

## Abstract

Background: The choice of prosthesis for aortic valve replacement (AVR) remains challenging. The risk of anticoagulation complications vs. the risk of aortic valve reintervention should be weighed. This study compared the outcomes of bioprosthetic vs. mechanical AVR in patients older and younger than 50. Methods: This retrospective study was conducted from 2009 to 2019 and involved 292 adult patients who underwent isolated AVR. The patients were divided according to their age (above 50 years or 50 years and younger) and the type of valves used in each age group. The outcomes of bioprosthetic valves (Groups 1a (>50 years) and 1b (≤50 years)) were compared with those of mechanical valves (Groups 2a (>50 years) and 2b (≤50 years)) in each age group. Results: The groups had nearly equal rates of preexisting comorbidities except for Group 1b, in which the rate of hypertension was greater (32.6% vs. 14.7%; *p* = 0.025). This group also had higher rates of old stroke (8.7% vs. 0%, *p* = 0.011) and higher creatinine clearance (127.62 (108.82–150.23) vs. 110.02 (84.87–144.49) mL/min; *p* = 0.026) than Group 1b. Patients in Group 1a were significantly older than Group 2a (64 (58–71) vs. 58 (54–67) years; *p* = 0.002). There was no significant difference in the NYHA class between the groups. The preoperative ejection fraction and other echocardiographic parameters did not differ significantly between the groups. Re-exploration for bleeding was more common in patients older than 50 years who underwent mechanical valve replacement (*p* = 0.021). There was no difference in other postoperative complications between the groups. The groups had no differences in survival, stroke, or bleeding rates. Aortic valve reintervention was significantly greater in patients ≤ 50 years old with bioprosthetic valves. There were no differences between groups in the changes in left ventricular mass, ejection fraction, or peak aortic valve pressure during the 5-year follow-up. Conclusions: The outcomes of mechanical and bioprosthetic valve replacement were comparable in patients older than 50 years. Using bioprosthetic valves in patients younger than 50 years was associated with a greater rate of valve reintervention, with no beneficial effect on the risk of bleeding or stroke.

## 1. Introduction

Although several studies have evaluated aortic valve replacement (AVR) outcomes using different valve prostheses, the valve choice remains challenging [1,2,3,4,5,6]. The risk of bleeding associated with anticoagulation therapy and possible teratogenic risk for women of childbearing age are two obstacles to consider before deciding to implant a mechanical valve [7,8]. Mechanical aortic valves were associated with greater bleeding rates than bioprosthetic valves in patients aged 50 to 69 years [9], consistent with studies on mechanical mitral valves [9]. Regarding bioprosthetic valve selection, structural valve deterioration and lower durability requiring reoperation are among the most feared drawbacks [10] despite the acceptable five-year durability of bioprosthetic AVR [11]. Earlier studies showed similar survival rates with mechanical or bioprosthetic AVR [12].

On the other hand, recent studies reported similar risks of bleeding and stroke between mechanical and bioprosthetic valves, with better long-term survival in patients with mechanical valves [8]. Recently, the introduction of new generations of bioprosthetic aortic valves with comparable costs to mechanical valves [13] and transcatheter aortic valve interventions [14] has increased the popularity of bioprosthetic valves. Moreover, lower anticoagulation intensity in patients with mechanical aortic valves might promote mechanical valve use in older patients [15].

Despite recent advances in aortic valve interventions through transcatheter and minimally invasive surgery [14,16,17,18], choosing an aortic valve prosthesis is challenging in young and old patients. Patients with rheumatic valve disease might require valve replacement at a young age. Bioprosthetic valves have the advantage of preventing anticoagulation during pregnancy; however, the risk of multiple reoperations is substantial. Therefore, further studies are required to explore the long-term differences between the two types of valves, especially in our Middle Eastern population, which has a high incidence of rheumatic heart disease requiring surgery at a young age. In this study, we aimed to investigate long-term outcomes in adult patients older or younger than 50 years after AVR with mechanical or bioprosthetic valves. Furthermore, we characterized patients who received mechanical and bioprosthetic valves in patients older or younger than 50 years.

## 2. Methods

### 2.1. Study Design and Patients

We conducted a retrospective cohort study that included 292 patients who underwent isolated AVR from 2009 to 2020 at Prince Sultan Cardiac Center, Riyadh, Saudi Arabia. We grouped the patients according to their age (≤50 years and >50 years) and type of implanted aortic valve (mechanical or bioprosthetic aortic valve). The groups included patients older than 50 years with either bioprosthetic (Group 1a: n = 111) or mechanical AVR (Group 2a: n = 38) and patients aged ≤50 years with bioprosthetic (Group 1b: n = 48) or mechanical AVR (Group 2b: n = 95). Patients who underwent AVR and concomitant coronary artery bypass grafting (CABG), mitral or tricuspid valve surgery, or other cardiac procedures were excluded from this study. Age older than 50 is the recommended age for the possibility of using bioprosthetic valves in the aortic position [19]. This study included patients with primary or redo AVR because this could be a factor that affected prostheses choice. 

Approval for this study was obtained from the Institutional Review Board (IRB approval No. 1676), and the need for patient consent was waived.

### 2.2. Study Data and Outcomes

Demographic, clinical, laboratory, and echocardiographic data were collected from paper and electronic medical charts. Preoperative data included age, body mass index (BMI), sex, EuroSCORE II, New York Heart Association class (NYHA), hypertension, diabetes mellitus (DM), chronic lung disease, renal impairment, liver disease, previous myocardial infarction (MI), previous heart failure hospitalization within one year prior to surgery, old stroke, atrial fibrillation (AF), previous cardiac surgery, previous percutaneous coronary intervention (PCI), previous transcatheter aortic valve replacement, hemoglobin level, and creatinine clearance. Operative data included emergency status (critical aortic stenosis or stuck aortic prosthesis), cardiopulmonary bypass (CPB), and cross-clamp times. Postoperative outcomes included hospital mortality, return to the operating room for bleeding, blood transfusion, early permanent pacemaker insertion (PPM), new-onset AF, stroke, MI, creatinine level (highest before discharge), respiratory failure, length of ICU, and length of hospital stay. The long-term outcomes were mortality, stroke, need for valve reintervention, bleeding, and cardiac rehospitalization for heart failure.

### 2.3. Outcomes

The primary endpoint was all-cause mortality during hospitalization or at follow-up. The secondary endpoints were major adverse events in the hospital and follow-up. Other secondary outcomes included all bleeding, stroke, and aortic valve reintervention.

### 2.4. Echocardiography

All patients underwent transthoracic echocardiograms before surgery and at discharge. Changes in the ejection fraction (EF), peak aortic valve pressure, and LV mass were reported and compared between the groups.

### 2.5. Prosthesis Types

The mechanical valves used in our study were St. Jude mechanical valves (SJM; St. Jude Medical Inc.; Minneapolis, MN, USA), ATS (Medtronic; Minneapolis, MN, USA), Carbomedics Sorin (LivaNova PLC, Sorin, Sluggia, Italy), and On-X (CryoLife, Kennesaw, GA, USA). Tissue valves were Magna valves (Edwards Lifesciences, Irvine, CA, USA), Trifecta (Abbott, Plymouth, MN, USA), Perceval sutureless valves (LivaNova Group, Milan, Italy), and Hancock (Medtronic Inc.; Minneapolis, MN, USA).

### 2.6. Follow-Up

All patients were clinically followed up in our outpatient clinic at 1, 6, and 12 months postoperatively and at yearly intervals thereafter. The patient’s vital status was confirmed by phone in June 2021 and by reviewing the medical records for all patients in June 2023. The hospital anticoagulation protocol was the same for mechanical and bioprosthetic valves. Patients were discharged on warfarin after achieving the target INR. and followed up in the anticoagulation clinic. The target INR was 2–3 for the mechanical valves for life, and patients with bioprosthetic valves had warfarin for only three months. Patients with bioprosthetic valves and contraindications to warfarin were discharged on aspirin only. 

### 2.7. Definitions

Hospital outcomes were defined as those occurring 30 days after surgery or within the same hospital admission. Preoperative variables were collected according to EuroSCORE II definitions [20].

### 2.8. Statistical Analysis

We used Stata 18 (Stata Corp, College Station, TX, USA) to perform all the statistical analyses. The Shapiro–Wilk test was used to test the distribution of the continuous variables. Normally distributed data are expressed as the mean and standard deviation and were compared with Student’s t-test. Nonnormal data are expressed as medians (25th–75th percentiles) and were compared with the Mann–Whitney test. Categorical data are presented as frequencies and percentages and were compared with Fisher’s exact test if the expected frequency was less than five. Survival distribution was plotted using Kaplan–Meier curves and compared with the log-rank test. The random effect model was used to compare the repeated measures among groups. A two-sided *p*-value of less than 0.05 was considered to indicate statistical significance.

## 3. Results

### 3.1. Baseline and Operative Characteristics

A greater proportion of male participants were in Group 2b (*p* = 0.018). The groups had no significant difference in body mass index (BMI). The EuroSCORE II was significantly greater in Group 2a than in Group 2b (0.94% vs. 0.87%, *p* = 0.049). The prevalence of hypertension and old stroke was significantly greater in Group 2a than in Group 2b. The groups had nearly equal rates of other preexisting comorbidities, with no significant differences observed between the groups in terms of smoking status, renal impairment status, diabetes status, liver disease status, chronic lung disease status, previous MI status, previous heart failure hospitalization status, AF status, previous cardiac surgery status, previous PCI status or previous transcatheter aortic valve intervention (TAVI) status. Patients in Group 1a had significantly lower hemoglobin levels and creatinine clearance levels than those in Group 2b.

Moreover, the groups had similar presentations, with no significant difference in the NYHA class (Table 1). There was no difference in the operative status between patients who underwent AVR tissue or mechanical surgery, regardless of their age group. The groups had no difference in cardiopulmonary bypass and ischemic times (Table 1).

### 3.2. Echocardiographic Characteristics

There was no difference in the preoperative ejection fraction between the groups. Ventricular mass and peak velocity did not vary significantly among the presented groups. Patients had similar end-diastolic and end-systolic diameters. There was no significant difference between participants regarding pulmonary artery systolic pressure, the severity of aortic valve regurgitation, or the severity of aortic valve stenosis (Table 2).

### 3.3. Postoperative Complications

Re-exploration for bleeding was more common in patients older than 50 years who underwent mechanical AVR (Group 2a) (*p* = 0.045). There was no difference in other postoperative complications between the groups. The hospital mortality rate was not significantly different among the studied groups. There were 4 deaths (3.6%) in Group 1a and 2 (5.26%) in Group 2a. However, there were no deaths among patients who were 50 years of age or younger and who underwent aortic valve replacement with a tissue valve, while there were no cases (2.11%) of deaths among patients who underwent mechanical valve replacement (*p* = 0.551) (Table 3).

### 3.4. Survival Rates

**Figure 1:**
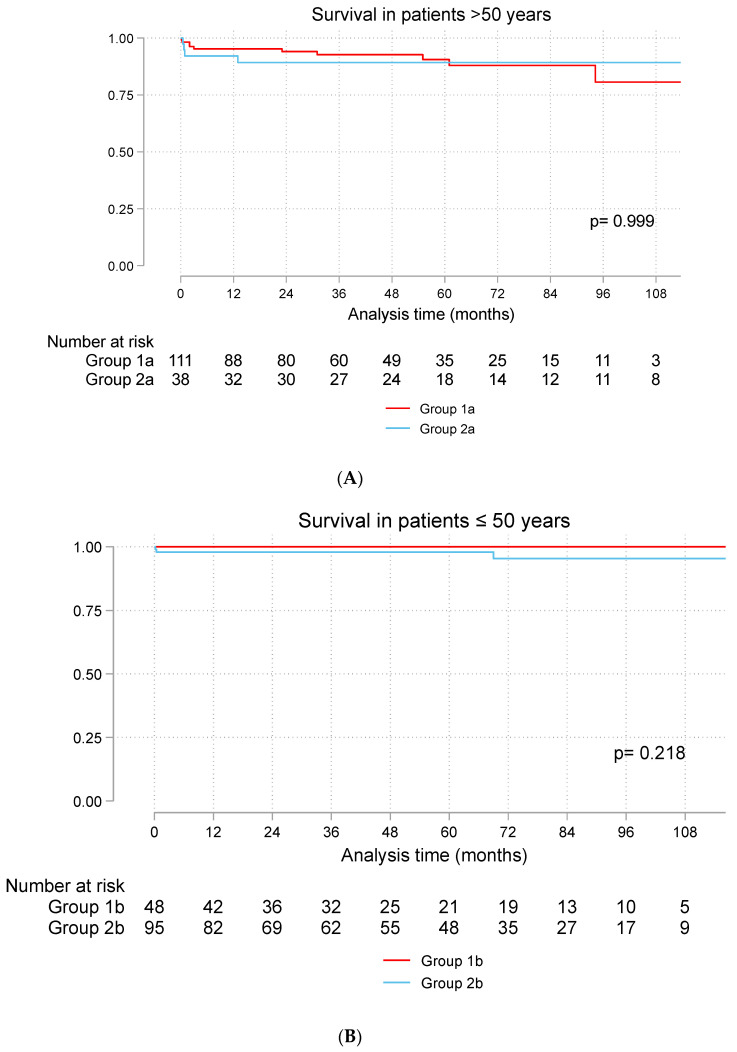
(**A**): Survival of patients > 50 years and (**B**): Survival of patients ≤ 50 years. Group 1a (bioprosthetic valve and age > 50 years), Group 2a (mechanical valve and age > 50 years), Group 1b (bioprosthetic valve and age ≤ 50 years), Group 2b (mechanical valve and age ≤ 50 years).

The median follow-up time was 45 months (22–72) for patients > 50 years. Survival at 5 and 10 years was 88% and 81% in Group 1a and 89% and 89%, respectively, in Group 2b. The total mortality rate was 9% (10 patients) in Group 1a and 10.5% (4 patients) in Group 2a. The median follow-up in patients ≤ 50 years was 56 (19–85) months. Survival in Group 1b was 100%, and that in Group 2b was 98% at 5 years and 95% at 10 years (Figure 1A,B).

### 3.5. Freedom from Reintervention

**Figure 2:**
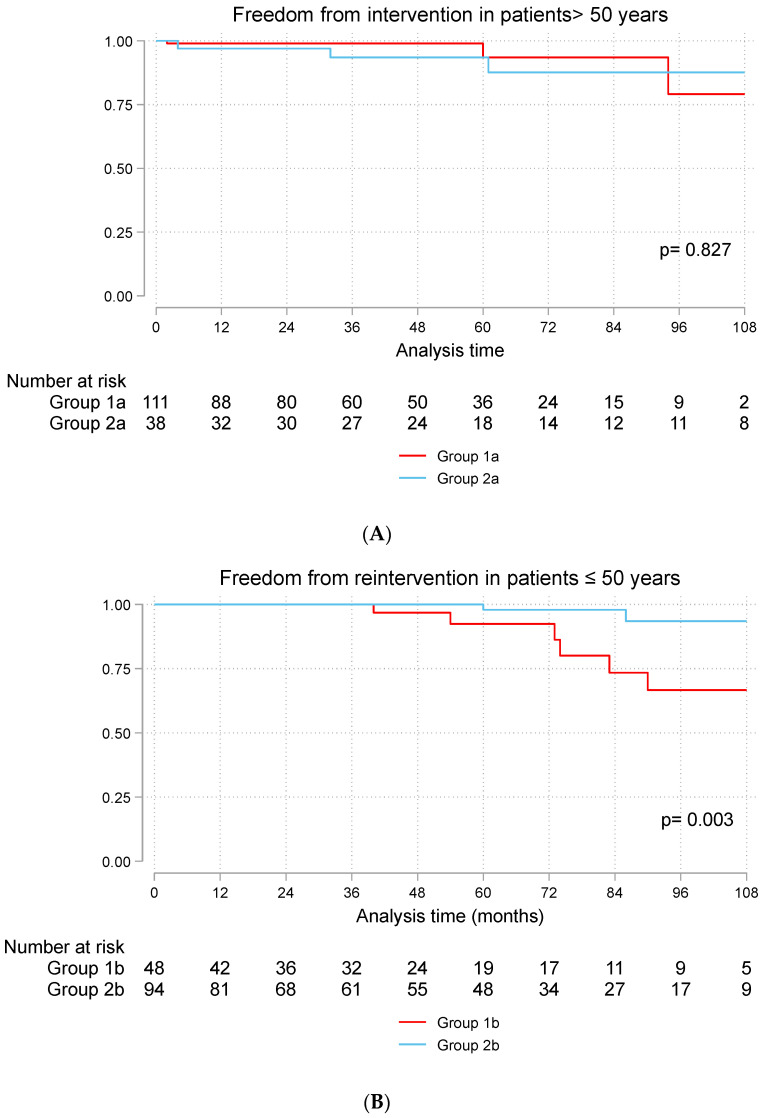
(**A**) Freedom from aortic valve reintervention in patients > 50 years and (**B**) in patients ≤ 50 years. Group 1a (bioprosthetic valve and age > 50 years), Group 2a (mechanical valve and age > 50 years), Group 1b (bioprosthetic valve and age ≤ 50 years), Group 2b (mechanical valve and age ≤ 50 years).

The rate of freedom from valve-related reintervention was 94% in Groups 1a and 2a after 5 years. Six patients had reintervention in Group 1a vs. three patients in Group 2a. At 5 years, 92% of the patients in Group 1b and 98% of the patients in Group 2b were free from reintervention (Figure 2A,B). Reintervention occurred in seven patients in Group 1b vs. four patients in Group 2b. Reintervention was performed surgically in 16 patients and transcatheter aortic valve replacement was performed in 4 cases. The causes of reinterventions were infective endocarditis (n = 4), degenerated prosthesis (n = 11), paravalvular leak (n = 3), and patient-prosthesis mismatch (n = 2). 

### 3.6. Freedom from Stroke

**Figure 3:**
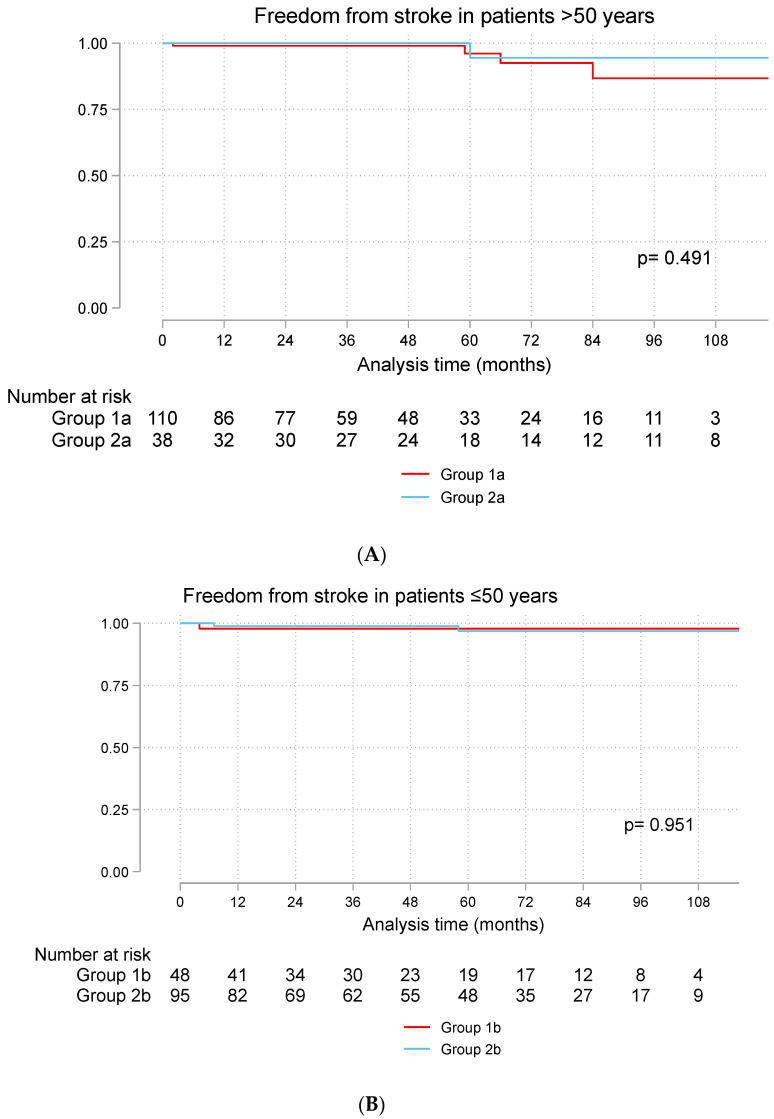
Freedom from stroke in patients > 50 years (**A**) and ≤50 years (**B**). Group 1a (bioprosthetic valve and age > 50 years), Group 2a (mechanical valve and age > 50 years), Group 1b (bioprosthetic valve and age ≤ 50 years), Group 2b (mechanical valve and age ≤ 50 years).

There were five strokes in Group 1a and one stroke in Group 2a. The prevalence of freedom stroke was 87% at 10 years in Group 1a and 94% in Group 2a. Stroke occurred in one patient in Group 1b and two in Group 2b. In both groups, the rate of freedom from stroke was 98% at 10 years (Figure 3A,B).

### 3.7. Freedom from Bleeding

**Figure 4:**
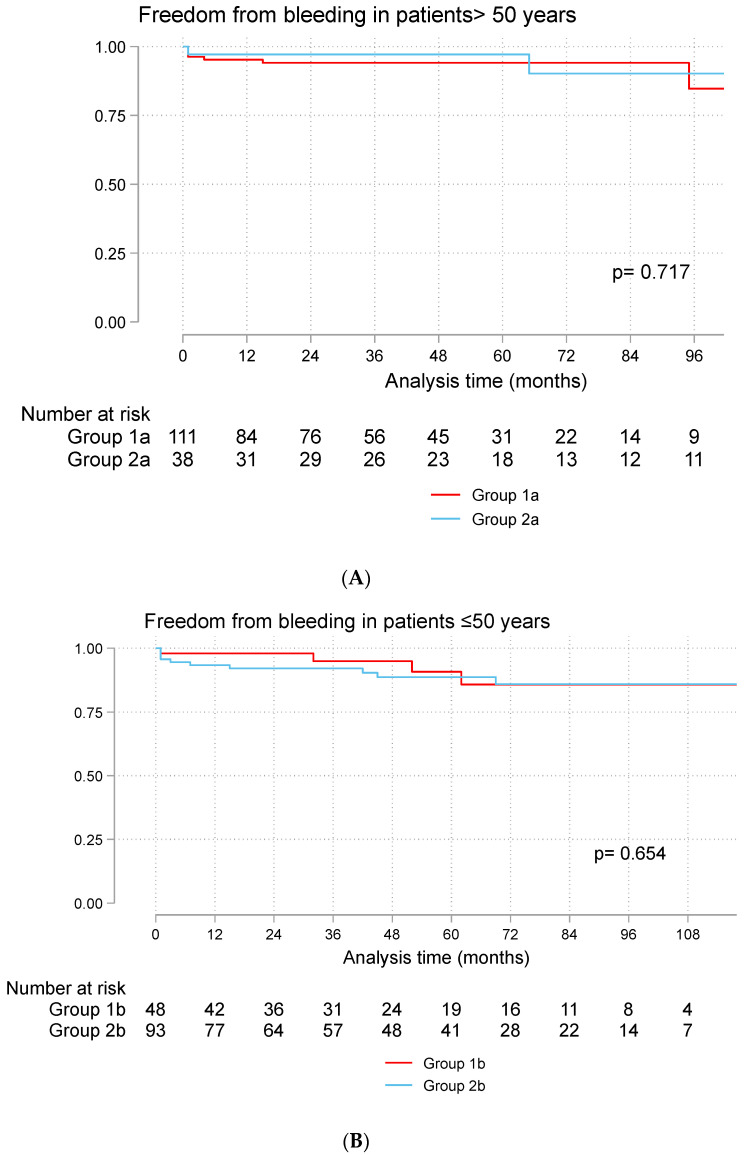
Freedom from bleeding in patients > 50 years (**A**) and ≤50 years (**B**). Group 1a (bioprosthetic valve and age > 50 years), Group 2a (mechanical valve and age > 50 years), Group 1b (bioprosthetic valve and age ≤ 50 years), Group 2b (mechanical valve and age ≤ 50 years).

There were seven bleeding episodes in Group 1a and 3 in Group 2a. There was 85% freedom from bleeding at 10 years in Group 1a and 77% in Group 2a. Four bleeding episodes occurred in Group 1b, and 12 occurred in Group 2b. The rate of freedom from bleeding was 86% at 10 years in both groups (Figure 4A,B).

### 3.8. Changes in Echocardiography

**Figure 5:**
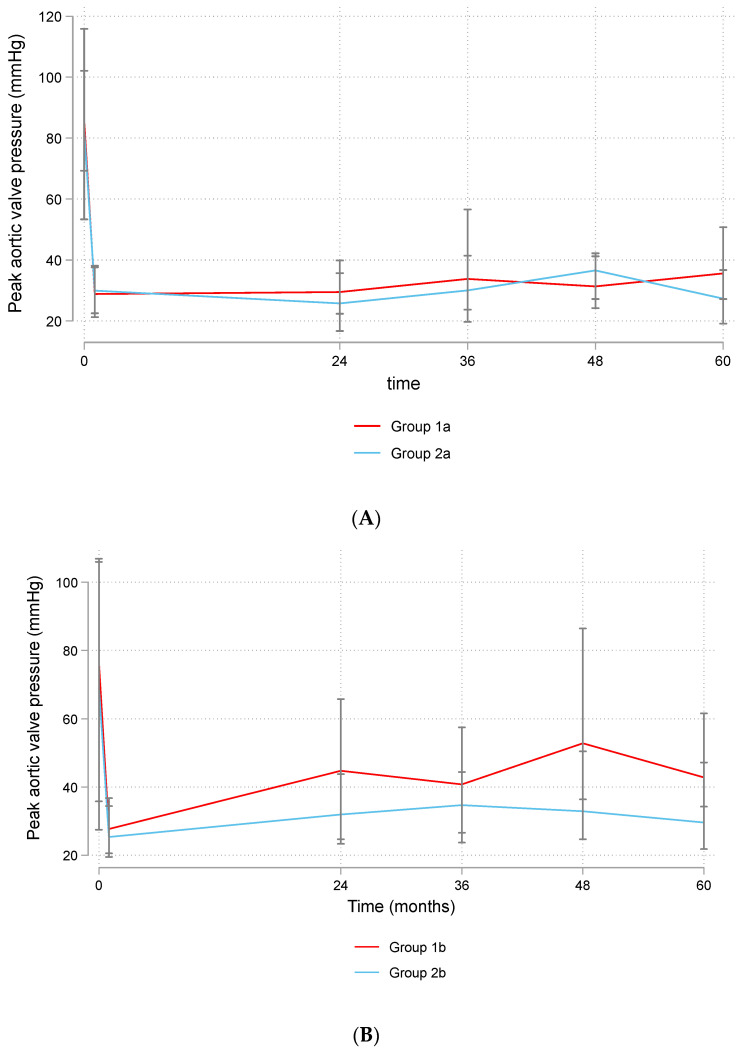
Changes in peak aortic valve pressure in patients > 50 years (**A**) and ≤50 years (**B**). Group 1a (bioprosthetic valve and age > 50 years), Group 2a (mechanical valve and age > 50 years), Group 1b (bioprosthetic valve and age ≤ 50 years), Group 2b (mechanical valve and age ≤ 50 years).

There was a significant reduction in the LV mass in Groups 1a and 2a over follow-up (β: −0.47 (95% CI: −0.60–−0.34), *p* < 0.001), with no difference between them (β: −6.86 (95% CI: −18.58–4.87), *p* = 0.252). The changes in ejection fraction were not significant (β: 0.02 (95% CI: −0.003–0.05), *p* = 0.08), and there was no difference between the groups (β: 2.66 (95% CI: −0.20–5.52), *p* = 0.068). The peak AV pressure significantly reduced during follow-up (β: −0.51 (95% CI: −0.66–−0.37), *p* < 0.001), with no difference between Groups 1a and 2a (β: −0.93 (95% CI: −8.23–6.36), *p* = 0.802) (Figure 5A).

Left ventricular mass decreased significantly over time (β: −0.72 (95% CI: −0.99–−0.44), *p* < 0.001), with no difference between Groups 1b and 2b (β: 2.39 (95% CI: −9.27–14.04), *p* = 0.688). There was a significant improvement in EF over time (β: 0.05 (95% CI: 0.02–0.08), *p* < 0.001), while there was no difference between Groups 1b and 2b (β: −0.73 (95% CI: −3.41–1.97), *p* = 0.597). There was a reduction in the peak AV pressure, but it did not reach a significant level (β: −0.15 (95% CI: −0.31–0.004), *p* = 0.056), and there was no difference between Groups 1b and 2b (β: −6.80 (95% CI: −14.58–0.98), *p* = 0.087) (Figure 5B).

## 4. Discussion

The challenges of choosing an aortic valve prosthesis are continuously debated. This study explored the differences in short- and long-term outcomes after AVR between patients who received bioprosthetic and those who received mechanical valves stratified by age. There was no difference in postoperative complications between groups apart from greater re-exploration for bleeding in patients > 50 years old with mechanical valves. The groups had no differences in survival, stroke, or bleeding rates. Aortic valve reintervention was significantly greater in patients ≤ 50 with bioprosthetic valves. There were no differences between groups in the changes in left ventricular mass, ejection fraction, or peak aortic valve pressure during the 5-year follow-up.

Male patients constituted the vast majority of the participants in all groups; however, the proportion of males was lower in patients ≤ 50 years of age who received bioprosthetic valves. This finding could be attributed to the increased use of bioprosthetic valves in women of childbearing age [21]. A meta-analysis conducted by Grashuis and colleagues showed that bioprosthetic valves for females of childbearing age with plans for future pregnancies were preferred over mechanical valves even when considering the possibility of receiving safe continuous low-dose oral anticoagulation during pregnancy [22]. In this study, the rate of preoperative comorbidities did not vary between the groups except for the rates of hypertension and old stroke, which were greater in patients ≤ 50 years old and who underwent bioprosthetic AVR. This finding could be attributed to the tendency to avoid the risk of anticoagulation in those patients. There were no differences in hospital complications among groups; however, re-exploration for bleeding was higher in older patients with mechanical valves. Not all patients with bioprosthetic valves had warfarin postoperatively, which could explain the higher bleeding rate in older patients with mechanical valves.

The long-term use of aortic valve bioprostheses at a young age is controversial. Malvindi and colleagues conducted research comparing bioprosthetic and mechanical AVR in patients between 50 and 69 years old. They reported similar survival, greater bleeding with mechanical valves, and greater reintervention with bioprosthetic valves [23]. Chiang and colleagues reported a greater cumulative incidence of bleeding following mechanical valve replacement than following bioprosthetic valve replacement in patients aged 50–69 years, with greater reintervention with bioprosthetic valves and similar survival and stroke rates [24]. Traxler and associates compared the long-term outcomes of patients aged between 50 and 69 years with bioprosthetic and mechanical valves. They reported a lower risk of bleeding, better survival, and a lower risk of myocardial infarction in patients with mechanical valves [25]. On the other hand, the risk of stroke was similar between the two valves. Kyto and colleagues matched patients with mechanical and bioprosthetic valves aged 50 to 70. They reported lower mortality, reoperation, and infective endocarditis in patients with mechanical valves [26]. Zhao and colleagues performed a meta-analysis of studies comparing mechanical and bioprosthetic AVR in patients aged >50 years [27]. They reported similar survival rates but reduced bleeding and greater structural valve deterioration with bioprosthetic valves. These studies indicate that expanding the indications for bioprosthetic valves in patients aged >50 years should be performed with caution. The risk of reoperation is still high; however, the advancement of transcatheter aortic valve replacement has mitigated the risk of surgical reoperation [28]. Our study demonstrated comparable reintervention and survival rates between both prostheses in patients > 50 years; moreover, there was no increased risk of bleeding or stroke in patients with mechanical valves.

Few studies have reported data on aortic valve prostheses in patients ≤ 50 years. Hirji and colleagues reported comparable mid-term and long-term bleeding and survival in patients with mechanical and bioprosthetic AVR aged 50 years and younger; however, the risk of reoperation was greater with bioprosthetic valves [29]. Similar findings were reported in other studies [10,30]. A study by Corona and colleagues reported similar structural bioprosthetic aortic valve deterioration in young and old patients [31]. Our study reported comparable survival, bleeding, and stroke rates in patients aged 50 years and younger; however, aortic valve reintervention remains an issue with bioprosthetic valves.

In summary, our study demonstrated that bioprosthetic and mechanical valves could be viable options for patients older than 50 years; however, bioprosthetic valves should be used with caution in younger patients because of the greater risk of reintervention.

### Study Limitations

This study has limitations. First, the sample size was relatively small, with an uneven distribution across the different groups. These differences could be attributed to the current recommendations for aortic valve prostheses. Second, this was a single-center study, and using anticoagulation protocols and follow-up could affect the outcomes. Third, the study is retrospective, with inherent referral and selection biases. Fourth, the valve type could have affected the outcomes, which were not evaluated in this study. Finally, the long-term follow-up is limited. Most reinterventions for bioprosthetic valve degeneration occur after 10 years and not all patients in our study completed 10 years. Studies with longer follow-ups beyond 10 years are recommended. 

## 5. Conclusions

Aortic valve replacement can be performed safely in adult patients using both biological and prosthetic valves, as they showed similar early survival rates. The outcomes of mechanical and bioprosthetic valves were comparable in patients older than 50 years. Using bioprosthetic valves in patients younger than 50 years was associated with a greater rate of valve reintervention, with no beneficial effect on the risk of bleeding or stroke.

## Figures and Tables

**Table 1:** Comparison of the preoperative patients’ baseline characteristics between aortic valve replacement using bioprosthetic and mechanical valves in patients aged >50 years and ≤50 years.

Variables	Group 1a (n = 111)	Group 2a (n = 38)	*p*-Value	Group 1b (n = 48)	Group 2b (n = 95)	*p*-Value
Male	81 (72.97%)	24 (63.16%)	0.304	28 (58.33%)	85 (89.47%)	0.018 *
Age, years	64 (58, 71)	58 (54, 67)	0.002 *	38.5 (28, 47.5)	31 (20, 43)	<0.001 *
BMI, kg/m^2^	29.30 (24.13, 3.73)	30.75 (26.50, 34.40)	0.150	25.42 (20.50, 32.26)	25.60 (19.46, 29.65)	0.891
EuroSCORE II, %	1.19 (0.87, 2.08)	1.2 (0.69, 2.05)	0.883	0.94 (0.69, 2.03)	0.87 (0.67, 1.52)	0.049 *
Risk factors						
Smoking	11 (10.58%)	6 (16.22%)	0.358	6 (13.33%)	12 (12.90%)	>0.99
Renal impairment	10 (9.35%)	7 (20%)	0.130	6 (13.4%)	3 (3.16%)	0.058
Hypertension, n	73 (66.97)	29 (78.38)	0.219	15 (32.61%)	14 (14.74%)	0.025 *
Diabetes	55 (50.46)	25 (67.57)	0.086	10 (21.74%)	10 (10.64%)	0.121
Liver disease	2 (1.90%)	1 (2.78%)	>0.99	1 (2.17%)	1 (1.06%)	0.551
Chronic lung disease	14 (13.08%)	4 (11.6%)	>0.99	2 (4.55%)	1 (1.06%)	0.238
Previous MI	2 (1.85%)	0	>0.99	0	1 (1.09%)	>0.99
Previous Heart Failure	1 (1.04%)	0	>0.99	1 (2.27%)	1 (1.14%)	>0.99
Old Stroke	6 (5.88%)	1 (2.78%)	0.676	4 (8.70%)	0	0.011
Atrial fibrillation	10 (9.52%)	3 (8.33%)	>0.99	1(2.17%)	5 (5.38%)	0.663
Previous Cardiac surgery	10 (9.26%)	8 (21.62%)	0.079	8 (17.02%)	22 (23.66%)	0.394
Previous PCI	6 (5.56%)	2 (5.41%)	>0.99	2 (4.26%)	0	0.110
Previous TAVI	4 (3.70%)	0	0.572	0	0	>0.99
Laboratory findings						
Hemoglobin (mg/dL)	13.5 (12, 14.7)	13.7 (11.8, 15)	0.858	13.1 (11.65, 14.75)	14.2 (13, 15.3)	0.016
Creatinine clearance (mL/min)	90.36 (62.90, 108.49)	101.45 (47.56, 124.45)	0.773	110.02 (84.87, 144.49)	127.62 (108.82, 150.23)	0.026 *
Symptoms						
NYHA class I II III IV	2 (1.98%) 22 (20.95%) 72 (68.57%) 9 (8.57%)	3 (7.89%) 7 (18.42%) 24 (63.16%) 4 (10.53%)	0.359	4 (8.33%) 12 (25%) 28 (58.33%) 3 (6.25%)	7 (7.37%) 34 (35.79%) 50 (52.63%) 4 (4.21%)	0.436
Emergency/urgent surgery	4 (3.88%)	2 (5.88%)	0.638	3 (6.38%)	4 (4.44%)	0.691
Cardiopulmonary bypass time, min	95 (79, 126)	94 (80, 122)	0.923	97 (84, 115)	98 (75, 115.5)	0.693
Cross clamp time, min	76 (61, 101)	75 (63, 101)	0.688	79 (65, 94)	74 (60, 98)	0.688

Continuous data are expressed as the mean and standard deviation if normally distributed or as the median and (Q1–Q3) if nonnormally distributed. Categorical data are expressed as numbers and percentages. Group 1a (bioprosthetic valve and age > 50 years), Group 2a (mechanical valve and age > 50 years), Group 1b (bioprosthetic valve and age ≤ 50 years), Group 2b (mechanical valve and age ≤ 50 years); BMI: body mass index; MI: myocardial infarction; NYHA: New York Heart Association. * Indicates a significant *p*-value (<0.05).

**Table 2:** Comparison of the preoperative echocardiographic data between aortic valve replacement using bioprosthetic and mechanical valves in patients aged >50 years and ≤50 years.

Variables	Group 1a (n = 111)	Group 2a (n = 38)	*p*-Value	Group 1b (n = 48)	Group 2b (n = 95)	*p*-Value
EF, %	55 (50, 60)	55 (55–60)	0.363	55 (50, 60)	55 (50–60)	0.492
Ventricular Mass (g/m^2^)	123.89 (98.5, 147.1)	104.85 (96.96, 139.54)	0.193	124.03 (92.9, 157.4)	133.48 (99.8, 169.86)	0.559
Peak velocity (m/s)	85.8 (69.3, 102.1)	82.55 (53.35, 115.85)	0.620	76.6 (35.8, 106.9)	69.4 (27.5, 106)	0.490
End-diastolic diameter (mm)	51 (46, 56)	50 (46, 55)	0.482	52 (47, 58)	54 (48, 62)	0.264
End-systolic diameter (mm)	34 (29, 39)	31.5 (28, 39.5)	0.418	36 (30, 43)	36 (30, 43)	0.937
Pulmonary artery systolic pressure (mmHg)	37 (30, 45)	30 (30, 40)	0.178	35 (30, 50)	30 (25, 40)	0.262
AR severity None Mild Moderate Moderately severe Severe	22 (22.92%) 33 (34.38%) 20 (20.83%) 5 (5.21%) 16 (16.76%)	10 (27.78%) 10 (27.78%) 8 (22.22%) 2 (5.56%) 16 (16.76%)	0.951	5 (11.36%) 5 (11.36%) 8 (18.60%) 1 (2.33%) 24 (55.81%)	9 (10.71%) 8 (9.52%) 16 (19.05%) 2 (2.38%) 49 (58.33%)	0.991
AS severity None Mild Moderate Severe	5 (5.21%) 3 (3.13%) 5 (5.21%) 83 (86.46%)	5 (14.29%) 0 (0%) 3 (8.57%) 27 (77.14%)	0.194	9 (21.95%) 6 (14.63%) 2 (4.88%) 24 (58.54%)	21 (28.38%) 9 (12.16%) 8 (10.81%) 36 (48.65%)	0.583

Continuous data are expressed as the mean and standard deviation if normally distributed or as the median and (Q1–Q3) if nonnormally distributed. Categorical data are expressed as numbers and percentages. Group 1a (bioprosthetic valve and age > 50 years), Group 2a (mechanical valve and age > 50 years), Group 1b (bioprosthetic valve and age ≤ 50 years), Group 2b (mechanical valve and age ≤ 50 years); EF: ejection fraction; AR: aortic regurgitation; AS: aortic stenosis.

**Table 3:** Comparison of postoperative data between aortic valve replacement using bioprosthetic and mechanical valves in patients aged >50 years or ≤50 years.

Variables	Group 1a (n = 111)	Group 2a (n = 38)	*p*-Value	Group 1b (n = 48)	Group 2b (n = 95)	*p*-Value
In-hospital mortality	4 (3.6%)	2 (5.26%)	0.645	0	2 (2.11%)	0.551
Re-exploration for bleeding	7 (6.73%)	7 (20%)	0.045 *	1 (2.13%)	5 (5.38%)	0.664
Blood transfusion	49 (50.52)	12 (37.50%)	0.226	18 (40.91%)	32 (36.36%)	0.704
Number of PRBCs	0 (0, 2)	0 (0, 2)	0.757	0 (0, 1)	0 (0, 1)	0.444
Early PPM	3 (2.88%)	1 (2.78%)	>0.99	0 (0%)	0 (0%)	>0.99
AF	13 (12.38%)	2( 5.41%)	0.354	1 (2.17%)	1 (1.08%)	>0.99
Stroke	0 (0%)	0 (0%)	>0.99	0 (0%)	1 (1.15%)	>0.99
MI	0 (0%)	0 (0%)	>0.99	0 (0%)	0 (0%)	>0.99
Highest creatinine, mmol/L	86 (74, 112)	83 (75, 124)	0.788	70 (53.5, 93.5)	74 (66.5, 88.5)	0.272
Respiratory failure	0 (0%)	1 (2.94%)	0.254	0 (0%)	0 (0%)	>0.99
ICU stay (d)	2 (1, 4)	1.5 (1, 4)	0.963	1(1, 2)	1 (1, 3)	0.297
Hospital stay duration (days)	8 (6, 13)	9 (6, 15)	0.572	7 (6, 9)	7 (6, 11)	0.905

Continuous data are expressed as the mean and standard deviation if normally distributed or as the median and (Q1–Q3) if nonnormally distributed. Categorical data are expressed as numbers and percentages. Group 1a (bioprosthetic valve and age > 50 years), Group 2a (mechanical valve and age > 50 years); Group 1b (bioprosthetic valve and age ≤ 50 years), Group 2b (mechanical valve and age ≤ 50 years); AF: atrial fibrillation; ICU: intensive care unit; MI: myocardial infarction; PPM: permanent pacemaker; PRBCs: packed red blood cells. * Indicates a significant *p*-value (<0.05).

## Data Availability

Data are contained within the article.
